# Simultaneous Onset of Acute Myeloid Leukemia and Decompensated Cirrhosis: A Rare Case

**DOI:** 10.7759/cureus.86990

**Published:** 2025-06-29

**Authors:** Wilfredo J Javier-Rojas, Alvin B Newman-Caro, Yizhi Lin, Carlos Montero

**Affiliations:** 1 Internal Medicine, HCA Florida Blake Hospital, Bradenton, USA; 2 Gastroenterology, HCA Florida Blake Hospital, Bradenton, USA

**Keywords:** acute myeloid leukemia (aml), aml, cirrhosis, decompensated liver cirrhosis, flow cytometry, hematologic malignancy, hereditary hemochromatosis (hh), iron overload

## Abstract

Acute myeloid leukemia (AML) is a hematologic malignancy marked by uncontrolled proliferation of abnormal myeloid precursor cells, while cirrhosis involves progressive hepatic fibrosis and architectural distortion. Despite differing pathophysiologies, both can present with overlapping clinical features, complicating the diagnostic process. We present the case of a 78-year-old female with a one-month history of bilateral lower extremity edema, dyspnea, fatigue, anorexia, low-grade fever, and progressive abdominal distension. Upon admission, a comprehensive metabolic panel revealed mild transaminitis, hyperbilirubinemia, and hypoalbuminemia. Complete blood count with differential revealed moderate leukocytosis, macrocytic anemia below the transfusion threshold, severe thrombocytopenia, and 10% circulating blasts. A contrast-enhanced computed tomography scan of the abdomen and pelvis showed cirrhotic liver morphology, portal hypertension, esophageal varices, splenomegaly with infarct, and minimal ascites. Chronic liver disease workup also indicated possible hereditary hemochromatosis. Hematologic evaluation, including peripheral blood flow cytometry, confirmed AML with 11.4% blasts expressing CD34, CD117, HLA-DR, CD33, and CD13. This case highlights the rare concurrent presentation of newly diagnosed AML and cirrhosis, emphasizing the diagnostic challenges posed by overlapping symptoms and the necessity of a multidisciplinary approach for timely identification and management.

## Introduction

Acute myeloid leukemia (AML) is a clonal hematopoietic stem cell malignancy characterized by the rapid proliferation and accumulation of immature myeloid precursors in the bone marrow, peripheral blood, and occasionally extramedullary tissues [1]. This proliferation disrupts normal hematopoiesis, leading to cytopenias such as anemia, thrombocytopenia, and neutropenia. AML typically presents with symptoms related to marrow failure, such as fatigue, infections, and bleeding tendencies, and requires timely recognition and management due to its aggressive clinical course [1].

Cirrhosis, in contrast, represents the end stage of chronic liver disease and is defined by diffuse hepatic fibrosis and the formation of regenerative nodules that replace normal liver architecture [2]. It results from prolonged hepatic injury due to various etiologies, including viral hepatitis, alcohol use disorder, and non-alcoholic fatty liver disease [2]. Hematologic abnormalities are common in cirrhosis and can arise from hypersplenism secondary to portal hypertension, chronic inflammation, or impaired hepatic synthesis of thrombopoietin [3]. Anemia and thrombocytopenia are particularly frequent [3], often attributed to splenic sequestration, bone marrow suppression, or gastrointestinal blood loss.

Despite their distinct pathophysiologies, AML and cirrhosis may present with overlapping clinical manifestations. In patients with known cirrhosis, cytopenias are frequently ascribed to hepatic dysfunction [3]; however, this assumption may delay the diagnosis of a possible concomitant hematologic malignancy. Therefore, when a complete blood count (CBC) reveals significant abnormalities, particularly the presence of abnormal cells or persistent/worsening cytopenias, especially in the absence of gastrointestinal bleeding or other identifiable causes, a thorough diagnostic evaluation is warranted. This should include consideration of primary bone marrow disorders such as AML, even in patients with a pre-existing or newly diagnosed cirrhosis.

The literature includes several case reports of patients with pre-existing cirrhosis who were subsequently diagnosed with AML [4]. Nevertheless, there are no documented instances of concurrent new-onset AML and new-onset decompensated liver cirrhosis. This case illustrates a complex clinical scenario, necessitating a multifaceted diagnostic approach to identify the coexisting, newly developed pathologies.

## Case presentation

A 78-year-old White female patient with a medical history of iatrogenic hypothyroidism secondary to total thyroidectomy presented with a one-month history of fluctuating bilateral lower extremity swelling, progressive dyspnea, fatigue, anorexia, intermittent low-grade fevers, and increasing abdominal distension. The lower extremity swelling fluctuated throughout the day, was exacerbated by prolonged standing, and was characterized by 2+ pitting edema on examination. Dyspnea initially occurred with exertion but gradually progressed to the point of limiting minimal activity. Fatigue was persistent and debilitating, significantly impairing her ability to perform daily tasks. She reported marked anorexia with early satiety and experienced an unintentional weight loss of approximately six pounds. The fevers were low-grade, peaking at 100.8°F, intermittent in nature, and primarily occurred in the evenings, accompanied by malaise and chills. Abdominal distension progressively worsened and was associated with bloating and discomfort. The patient denied any history of alcohol use, smoking, intravenous drug use, tattooing, or prior blood transfusions. She has been in a monogamous long-term relationship. Her family history was notable only for maternal essential hypertension, with no other known hereditary conditions. Her surgical history was significant for a total thyroidectomy performed in 1993 due to refractory Graves’ disease. On presentation, her vital signs were as follows: temperature 98.0°F, heart rate 94 beats per minute, respiratory rate 18 breaths per minute, blood pressure 99/61 mmHg, and oxygen saturation 95% on room air. A Confusion Assessment Method evaluation was negative for delirium. Physical examination revealed bilateral lower extremity edema (2+ pitting) and mild ascites.

Upon admission, a comprehensive metabolic panel (CMP) revealed a total bilirubin level of 1.8 mg/dL (reference range: 0.2-1.0 mg/dL), an aspartate aminotransferase (AST) level of 49 U/L (15-37 U/L), and hypoalbuminemia with an albumin level of 2.5 g/dL (3.4-5.0 g/dL). A CBC demonstrated significant abnormalities, including leukocytosis with a white blood cell count of 26.9 × 10³/µL (4.0-10.5 × 10³/µL), severe anemia with a hemoglobin level of 5.5 g/dL (11.2-15.7 g/dL), macrocytosis with a mean corpuscular volume of 123 fL (79.4-94.8 fL), and thrombocytopenia with a platelet count of 34 × 10³/µL (150-450 × 10³/µL). The CBC differential showed immature granulocytes at 9.4% (0.0-0.4%), neutropenia with neutrophils at 22.2% (34.0-71.1%), relative lymphopenia at 16.7% (19.3-51.7%), and a striking monocytosis at 49.2% (4.7-12.5%), findings that are markedly abnormal and suggest a possible underlying myeloid neoplasm. Coagulation studies revealed a prothrombin time (PT) of 17.1 seconds and an international normalized ratio (INR) of 1.6. All findings from the CMP, CBC with differential, and coagulation studies are detailed in Table 1. Based on the semiquantitative grading system analysis for peripheral blood smear findings (Table 2), additional CBC findings included macrocytosis (4+) with normocytic anemia, slight anisopoikilocytosis (3+), slight target cells (2+), dacrocytes (1+), schistocytes (1+), smudge cells (1+), leukocytosis with 10% blasts, thrombocytopenia, and giant platelets, without platelet aggregates (Table 3).

**Table 1:** Summary of laboratory findings, including the CMP, CBC with differential, and coagulation studies APTT, activated partial thromboplastin time; ALT, alanine aminotransferase; ALP, alkaline phosphatase; AST, aspartate aminotransferase; BUN, blood urea nitrogen; CBC, complete blood count; CMP, comprehensive metabolic panel; INR, international normalized ratio; MCV, mean corpuscular volume; PT, prothrombin time; WBC, white blood cell

Test Category	Test	Value	Reference Range
CMP	Sodium	136 mmol/L	136–145
	Potassium	4.1 mmol/L	3.5–5.1
	Chloride	107 mmol/L	98–107
	Carbon dioxide	26.2 mmol/L	21.0–32.0
	BUN	20 mg/dL	7–18
	Creatinine	0.8 mg/dL	0.6–1.0
	Glucose	104 mg/dL	74–106
	Albumin	2.5 g/dL	3.4–5.0
	Total bilirubin	1.8 mg/dL	0.2–1.0
	AST	49 U/L	15–37
	ALT	30 U/L	12–56
	ALP	91 U/L	45–117
CBC	WBC count	26.9 × 10³/µL	4.0–10.5
	Hemoglobin	5.5 g/dL	11.2–15.7
	MCV	123 fL	79.4–94.8
	Platelet count	34 × 10³/µL	150–450
CBC differential	Immature granulocytes	9.4%	0.0–0.4
	Neutrophils	22.2%	34.0–71.1
	Lymphocytes	16.7%	19.3–51.7
	Monocytes	49.2%	4.7–12.5
Coagulation studies	PT	17.1 seconds	10.0–12.8
	INR	1.6	0.8–1.1
	APTT	29 seconds	25–38

**Table 2:** Semiquantitative grading system for peripheral blood smear findings

Grade	Description	Interpretation
1+	Rare or scant	Few cells seen per field
2+	Few	Slightly higher number of cells than 1+
3+	Moderate	Moderate number of cells observed
4+	Many or numerous	Numerous or heavy presence of cells

**Table 3:** Overview of CBC morphology and interpretation CBC, complete blood count

Test Category	Finding	Grade/Description	Notes
CBC morphology	Macrocytosis	4+ (numerous)	Associated with normocytic anemia
	Anisopoikilocytosis	3+ (moderate)	–
	Target cells	2+ (few)	–
	Dacrocytes	1+ (rare or scant)	–
	Schistocytes	1+ (rare or scant)	–
	Smudge cells	1+ (rare or scant)	–
CBC interpretation	Leukocytosis with blasts	10%	Suggestive of a possible underlying hematologic disorder
	Thrombocytopenia with giant platelets	Present	No platelet aggregates observed

**Figure 1:**
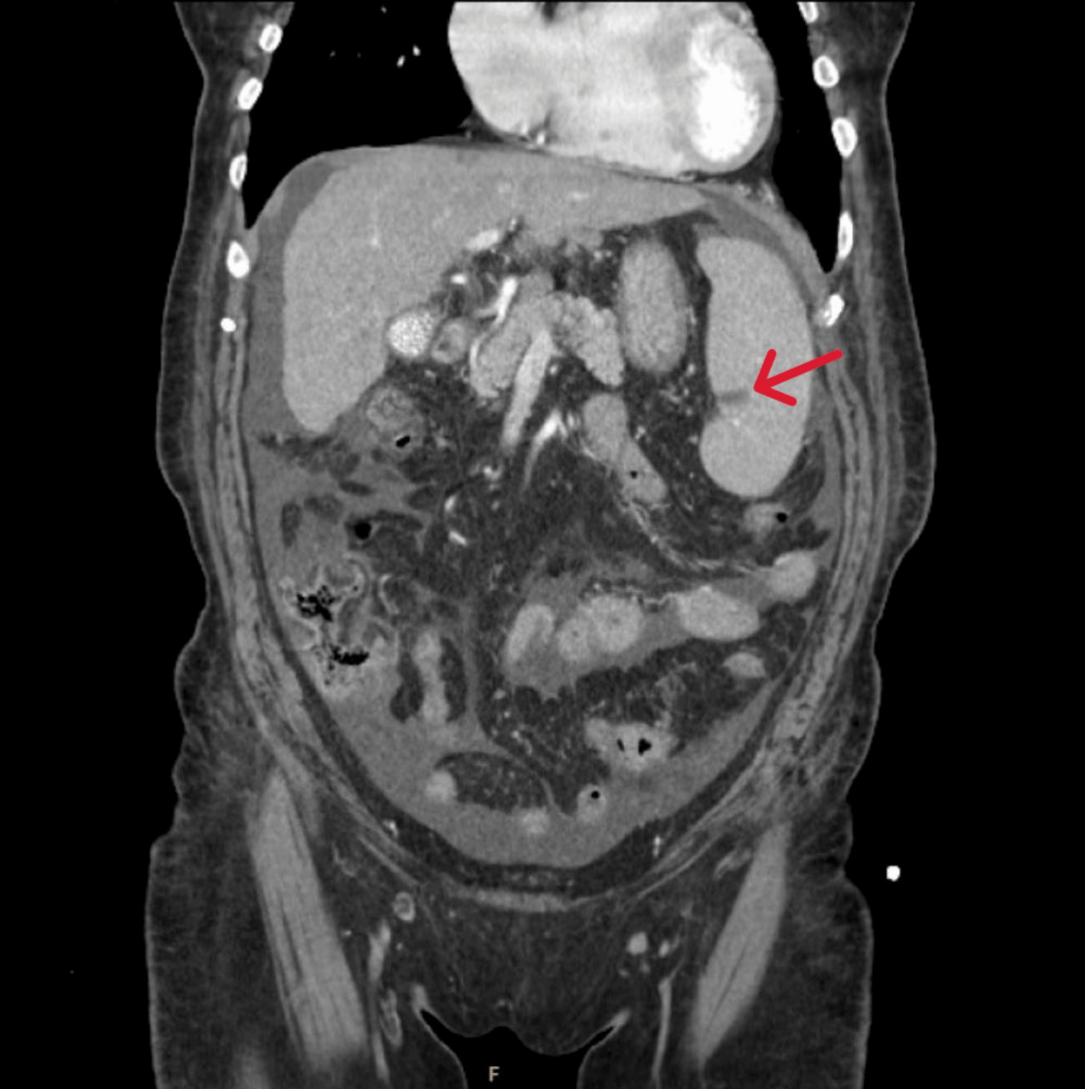
Contrast-enhanced computed tomography scan of the abdomen and pelvis Imaging revealed a small, nodular liver contour consistent with cirrhosis, mild splenomegaly measuring 13.33 cm with a focal infarct (red arrow), and minimal ascites. Although not visualized in this image, the portal, hepatic, and splenic veins were noted to be patent. The portal vein measured 13.8 mm in diameter. Features of portal hypertension were present, including large esophageal varices measuring 4.8 mm (not visualized in this image). Given the clinical context of acute myeloid leukemia, the splenic infarction is more likely attributable to leukemic infiltration and impaired splenic perfusion secondary to circulating leukemic blasts, rather than portal hypertension.

A contrast-enhanced computed tomography (CT) scan of the abdomen and pelvis demonstrated a small nodular liver consistent with cirrhosis, portal hypertension, large esophageal varices, mild splenomegaly (as per grading criteria, Table 4) with a small infarct, and minimal ascites (Figure 1). The patient's Model for End-Stage Liver Disease (MELD) score was 23, and the Child-Turcotte-Pugh was Class B, with 8 points. Doppler ultrasound of the bilateral lower extremities showed normal compression and augmentation, with subcutaneous soft tissue edema. The patient received two units of packed red blood cells and one unit of platelets. Given the presence of labile hypertension and esophageal varices, propranolol, with defined holding parameters, was initiated in place of carvedilol due to the patient’s high risk for circulatory dysfunction, particularly in the context of suspected underlying hematologic malignancy and fluid overload status. Therapy with proton pump inhibitors and octreotide was also commenced.

**Table 4:** Splenomegaly grading criteria

Grade of Splenomegaly	Craniocaudal Length (cm)	Clinical Context/Notes
Normal	≤13	Upper limit, general adult population [5]
Mild	>13–15	Above normal, not massive [5]
Moderate	>15–20	Intermediate enlargement [6]
Severe (massive)	≥20	"Massive" (≥17 cm), "supramassive" (≥22 cm) [6]

In the context of new-onset decompensated cirrhosis with transfusion-dependent anemia, a gastroenterology consultation was obtained. Esophagogastroduodenoscopy (EGD) revealed small, grade 1 esophageal varices without high-risk stigmata, gastritis, and mild portal hypertensive gastropathy with friable mucosa in the antrum, where biopsy showed reactive gastropathy. The hepatitis profile, antinuclear antibodies, anti-smooth muscle antibodies, and anti-mitochondrial antibodies were all negative. Serum ceruloplasmin and alpha-1 antitrypsin levels were within normal limits. Iron studies revealed an iron level of 158 µg/dL, total iron-binding capacity (TIBC) of 197 mcg/dL, transferrin saturation of 80%, and ferritin of 1390 ng/mL, suggestive of hereditary hemochromatosis (HH). Given the patient's history of iatrogenic hypothyroidism, Metabolic Dysfunction-Associated Steatotic Liver Disease (MASLD) was initially suspected. However, all prior thyroid-stimulating hormone levels were within normal limits, and the patient has no history of alcohol use, diabetes mellitus, hypertension, hyperlipidemia, or obesity. Given that MASLD is a diagnosis of exclusion, the absence of these risk factors makes it a less likely diagnosis in this case. Table 5 provides a summary of the chronic liver disease workup. Further details regarding the iron studies are summarized in Table 6.

**Table 5:** Findings from the laboratory evaluation of chronic liver disease

Test Category	Test	Value	Reference Range
Hepatitis profile	Hepatitis A IgM antibody	Negative	Negative
	Hepatitis B surface antigen	Negative	Negative
	Hepatitis B core IgM antibody	Negative	Negative
	Hepatitis C antibody	Negative	Negative
Autoimmune markers	Antinuclear antibodies	Negative	Negative
	Anti-smooth muscle antibodies	9.0 units	0.0–19
	Anti-mitochondrial antibodies	0.63 index	0.0–0.9
Metabolic markers	Serum ceruloplasmin	23.2 mg/dL	19.0–39.0
	Alpha-1 antitrypsin	182 mg/dL	101–187
Thyroid function	Thyroid-stimulating hormone	3.07 µIU/mL	0.36–3.74

**Table 6:** Detailed findings from the iron panel and supplementary hematologic and oncologic laboratory findings

Test Category	Test	Value	Reference Range
Iron studies	Iron level	158 µg/dL	35–150
	Total iron-binding capacity	197 µg/dL	260–445
	Iron saturation (transferrin)	80%	20–55
	Ferritin	1,390 ng/mL	8–388
Hematology/oncology laboratory tests	Cyanocobalamin (vitamin B12)	>1,000 pg/mL	211–911
	Folate (vitamin B9)	7.5 ng/mL	>2.76
	Uric acid	4.9 mg/dL	2.6–6.0
	Lactate dehydrogenase	536 U/L	87–241

**Figure 2:**
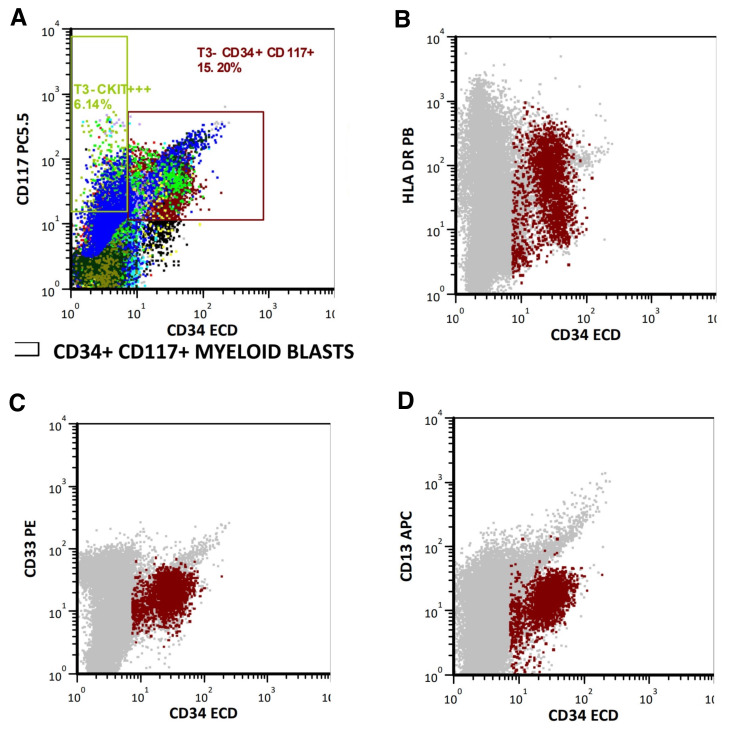
Findings of flow cytometry immunophenotyping Dual fluorescence scatter plots from peripheral blood demonstrating an increased population of circulating myeloid blasts (11.4% of cells), expressing CD34 (A), CD117 (A), HLA-DR (B), and subsets of CD33 (C) and CD13 (D), findings consistent with a diagnosis of acute myeloid leukemia.

Given the EGD findings, absence of overt bleeding, concern for hematologic malignancy, and evidence of left shift with blasts 10% on CBC, a hematology/oncology consultation was requested. Cyanocobalamin (vitamin B12) level was >1,000 pg/mL and folate (vitamin B9) level was 7.5 ng/mL, making vitamin deficiency an unlikely cause of the anemia (Table 6). JAK2 mutation polymerase chain reaction from peripheral blood was negative for polycythemia vera. BCR/ABL1 fluorescence in situ hybridization from peripheral blood showed normal probe signals, making chronic myeloid leukemia less likely. Flow cytometry immunophenotyping (Figure 2) from peripheral blood revealed increased circulating myeloid blasts (11.4% of cells), expressing CD34, CD117, HLA-DR, and subsets of CD33 and CD13, consistent with AML. Table 7 provides an overview of the flow cytometric analysis. Uric acid was 4.9 mg/dL and lactate dehydrogenase was 536 U/L (Table 6), indicating high tumor burden and rapid cell turnover.

**Table 7:** Flow cytometry alert conditions and results

Alert	Condition	Result
Kappa/lambda high	>6	1.32%
Kappa/lambda low	<0.5	1.32%
CD34, CD117 + myeloid blasts	>4	15.20%
Promyelocytes high	>20	3.00%
CKIT+ cells elevated	>5% of cells	6.14%
Increased monocytes	>20	18.69%
Increased blast gate	>5% of cells	2.61%
Increased immature monos	>10% of cells	3.90%
Myeloid blasts CD7+	>20% of blasts	2.14%
Myeloid blasts CD56+	>20% of blasts	1.29%
Myeloid blasts CD19+	>20% of blasts	3.86%
Myeloid blasts CD2+	>20% of blasts	0.54%
Plasma cells increased	>0.5% of cells	0.00%
Abnormal plasma cells	>50% abnormal	0.00%
CD3+ CD5- T cells	>5% of cells	0.45%
CD3+ DNT expanded	>10% of T cells	2.14%
CD3+ double positive expanded	>10% of T cells	1.14%
CD5+ B cells	>5% CD19+ CD5+	0.00%
CD10+ B cells	>5% CD19+ CD10+	0.00%
CD11c B cells	>5% CD19+ CD11c+	0.26%
CD56+ CD45-	>0.5%	0.00%
Increased eosinophils	>10%	5.47%
CD3+ CD7- T cells	>50% of T cells	7.24%
CD3+ CD2+ T cells	>90% of CD3+	99.19%

Based on the iron study findings, HH was considered a leading differential diagnosis for the patient’s newly diagnosed decompensated cirrhosis. However, definitive confirmation would require genetic testing and/or liver biopsy. Genotyping for HH could not be performed due to the time-sensitive nature of the patient's condition, as she was discharged shortly after the diagnosis of AML to initiate advanced care and chemotherapy. Outpatient follow-up was planned to include colonoscopy, with capsule endoscopy if indicated, as well as liver ultrasound elastography.

## Discussion

The diagnosis of AML is inherently challenging, and the complexity increases when it coexists with new-onset decompensated liver cirrhosis due to overlapping presentations. Common concurrent presentations include severe anemia, thrombocytopenia, prolonged PT/INR, elevated liver enzymes, hyperbilirubinemia, hypoalbuminemia, jaundice, fatigue, hepatosplenomegaly, encephalopathy, and renal insufficiency [4]. The presence of anemia, thrombocytopenia, or other cell-line abnormalities, regardless of the presence or absence of overt bleeding, should prompt consideration of an underlying hematologic malignancy as a potential cause, as demonstrated in our patient.

In our patient, although decompensated liver cirrhosis could have contributed to the anemia and thrombocytopenia, a thorough evaluation, including EGD, revealed no signs of high-risk stigmata or overt gastrointestinal bleeding. Given this, the most likely cause of the hematologic disturbances was then presumed to be a hematologic malignancy. Subsequent diagnostic analysis confirmed this suspicion.

When a peripheral smear and CMP reveal abnormalities, such as elevated liver enzymes, with or without corresponding physical findings, further evaluation is essential to differentiate between hematologic malignancy and liver disease [4]. The literature notes that some cases of AML can present with acute liver failure and obstructive jaundice [7,8]. However, it is equally important to consider primary liver disease in such scenarios. Therefore, timely and appropriate imaging is critical for accurate diagnosis and management.

In our patient, based on the results of the iron studies and the absence of other risk factors for cirrhosis, hemochromatosis remains a potential differential diagnosis. The findings, including elevated iron, iron saturation, and ferritin levels, alongside a low TIBC, are suggestive of this condition. Notably, the patient's iron saturation (transferrin) was 80%, which is a key diagnostic marker [9]. According to the literature, in individuals with C282Y homozygosity, a transferrin level greater than 45% identifies 97.9%-100% of cases, and a ferritin level exceeding 1,000 ng/mL (1,390 ng/mL in our patient) is associated with an increased risk of cirrhosis and other complications [9-11].

Iron overload in patients with AML or myelodysplastic syndrome can result from ineffective erythropoiesis and frequent red blood cell transfusions [12]. Nevertheless, based on the findings from the iron studies performed, hemochromatosis remains the most probable cause of the iron overload state in our patient. Iron overload has been linked to a poor prognosis in AML, as it contributes to oxidative stress, which promotes neoplastic cell proliferation and increases tumor burden [13] by upregulating oncogenes such as FOS [14]. Additionally, elevated ferritin levels are associated with reduced long-term survival in patients with AML [15].

Due to its association with oxidative stress, iron overload, regardless of etiology, may contribute to genomic instability, representing a potential risk factor for hematologic malignancies. However, this has not been shown to result in an increased incidence of AML in patients with HH [16].

## Conclusions

New-onset AML has been reported in patients with existing cirrhosis, but simultaneous new-onset of both conditions is undocumented, highlighting the novelty of this case. The concurrent development of AML and decompensated liver cirrhosis presents a complex diagnostic challenge due to overlapping symptoms. A multifactorial approach is essential for accurate diagnoses. Early intervention in both conditions is crucial for improving survival outcomes. Additionally, a thorough workup to identify the cause of cirrhosis, such as iron overload, can guide treatment strategies and improve prognosis in AML patients.
